# Demographic and Clinical Insights into Pediatric Asthma Exacerbations: A Retrospective Study at a Tertiary Care Center in the United Arab Emirates

**DOI:** 10.1055/s-0046-1824567

**Published:** 2026-06-23

**Authors:** Sinan Yavuz, Batool Zaffar Ali, Rana Salman, Mina Almanasir, Funda Cipe, Amal Sherif, Safiya Saif, Nujoud Al Zaabi, Maged Nabawi, Nader Francis

**Affiliations:** 1 Department of Pediatrics/Pulmonology Unit, Al Qassimi Women and Children Hospital, Sharjah, United Arab Emirates; 2 Department of Pediatrics, Al Qassimi Women and Children Hospital, Sharjah, United Arab Emirates; 3 Department of Pediatrics/Pediatric Intensive Care Unit, Al Qassimi Women and Children Hospital, Sharjah, United Arab Emirates

**Keywords:** asthma, asthma exacerbation, pediatric, severity

## Abstract

**Background:**

Asthma is a heterogeneous disease characterized by chronic airway inflammation, leading to airway hyperresponsiveness and recurrent episodes of wheezing, respiratory distress, chest tightness, and/or coughing.

**Objective:**

This study aims to describe the demographic characteristics, clinical presentation, management strategies, and outcomes of patients with asthma exacerbations.

**Methods:**

This retrospective study analyzed data from children aged 2 to 13 years who were admitted with a diagnosis of asthma exacerbation between January 2018 and July 2023. The study was conducted at a tertiary referral hospital providing inpatient care.

**Results:**

Univariate and multivariate analyses were performed to identify risk factors associated with severe asthma exacerbations (SAEs). Univariate analyses, including chi-square tests and simple logistic regression, showed that chest recession, respiratory distress, use of prophylactic medications, obesity, and a family history of allergy were significantly associated with severe exacerbations. However, obesity was associated with lower odds of developing SAE.

Comparisons of continuous variables using two-sample *t* -tests, Mann-Whitney U tests, and logistic regression revealed significant differences in age, respiratory rate, heart rate, and oxygen saturation between groups. Multiple logistic regression analyses, assessed using likelihood ratio tests, were then conducted to refine the prediction model. Increased respiratory rate, younger age, and lower oxygen saturation were identified as significant predictors of severe or life-threatening asthma exacerbations.

**Conclusion:**

Younger age, increased respiratory and heart rates, lower oxygen saturation, chest recession, respiratory distress, use of prophylactic medications, and a family history of allergy were identified as significant predictors of asthma exacerbation severity.

## Introduction

Asthma is a chronic inflammatory airway disease with a high prevalence, affecting approximately 10% of children and 5% of adults in the Western world. 1 2 In the United Arab Emirates (UAE), the prevalence of asthma among children aged 6 to 14 years ranges from 6 to 13%, with an overall rate of 7.4% in the population. 3 4 Asthma is a major cause of disability and healthcare utilization and significantly reduces quality of life. 5 This impact is particularly evident during asthma exacerbations, which substantially affect patients and their families.

According to the Global Initiative for Asthma (GINA) guidelines, “acute exacerbations” are defined as episodes characterized by a noticeable worsening of symptoms, including shortness of breath, coughing, wheezing, or chest tightness, either individually or in combination. These episodes are also associated with reduced airflow during exhalation, which can be measured using lung function tests. 6

To minimize asthma exacerbations, treatment is adjusted in a stepwise manner based on the patient's level of asthma control. 7 Management typically includes the daily use of a controller medication and the occasional use of short-acting bronchodilators for rapid symptom relief. Adherence to treatment is essential for achieving optimal therapeutic outcomes. 8 Poor or inconsistent adherence has been linked to adverse outcomes, including increased mortality, more frequent exacerbations, higher healthcare costs, and reduced quality of life. 5 6 7 8 9

Several factors, including country, age, sex, and ethnicity, influence adherence to asthma treatment, which is often suboptimal. 10 Low adherence rates are frequently attributed to safety concerns regarding inhaled corticosteroids (ICS), commonly referred to as “steroid phobia” among patients and caregivers. 11 The use of ICS has been associated with potential growth impairment in children and other systemic adverse effects. 8 Additionally, many ICS regimens require twice-daily administration, which may further reduce adherence compared with once-daily dosing. 12

Poor adherence to ICS has been associated with an increased risk of exacerbations, particularly in severe asthma cases. Severe asthma is defined as inadequate symptom control despite high-dose ICS treatment combined with additional controllers or oral corticosteroids for at least 6 months per year. Moderate asthma, by contrast, is characterized by daily symptoms, daily use of short-acting inhaled asthma medication, and nighttime symptoms occurring more than once per week but not every day. 13 Severe asthma in children accounts for only 2 to 5% of cases. Although these patients can often be managed with current therapies, they may remain difficult to treat (DTA). 14 Common findings include elevated total serum immunoglobulin E (IgE), increased blood eosinophil counts, and multiple aeroallergen sensitizations. 15 Bronchial hyperresponsiveness (BHR) and reduced lung function are also reported 15 16 17 18 ; however, existing literature presents conflicting evidence.

This retrospective study aims to review cases of asthma among children hospitalized at Al Qassimi Women and Children Hospital (AQWCH) over a 5-year period and to describe their demographic characteristics, clinical presentation, epidemiology, etiology, management strategies, and outcomes.

## Methods

### Study Design and Data Sources

This study is a retrospective chart review (RCR) conducted at AQWCH in the UAE. De-identified medical records of patients admitted with a history of asthma exacerbation between January 2018 and July 2023 were analyzed. The data source consisted of digital medical records of children diagnosed with asthma exacerbation during this period. Patients were identified using diagnosis codes and age ranges.

All selected patients were minors, and their data were anonymized and stored on password-protected computers accessible only to the principal investigator (PI). Ethical approval for this study was obtained from the Ministry of Health and Prevention Research Ethics Committee (MOHAP REC) (Approval Number: MOHAP/DXB-REC/S.O.N/No. 119/2023).

### Study Enrollment

Although this was a retrospective study, inclusion and exclusion criteria were applied to define the study population. Cases were identified using diagnosis codes and age filters.

The study included children aged 2 to 13 years who were admitted during the specified period with a final diagnosis of asthma exacerbation. Children presenting with their first asthma episode and those younger than 2 years or older than 13 years were excluded.

### Study Procedures

The study involved a review of existing medical records. A data abstraction form was used to collect information from digital records, including demographic information (nationality, age, gender, and duration of hospital stay), clinical features (fever, cough, respiratory distress, chest pain, chest recession, chest dullness, reduced air entry, crackles, wheezing, respiratory rate, heart rate, and oxygen saturation), severity assessment, admission status (pediatric intensive care unit [PICU] or pediatric ward), adherence to maintenance treatment, frequency of exacerbations in the past year, systemic steroid use, prior hospital admissions, family history of asthma, allergic rhinitis, eczema, exposure to smoking at home, prior PICU admissions, laboratory parameters (white blood cell [WBC] count, hemoglobin, mean corpuscular volume [MCV], C-reactive protein [CRP], total IgE, eosinophil count, vitamin D, iron level, ferritin level, transferrin level, total iron-binding capacity [TIBC], virology studies, and blood cultures), radiologic findings (chest X-ray), and treatment variables (systemic steroids, magnesium sulfate, intravenous salbutamol, and requirement for mechanical ventilation).

### Assessment of Severity Level

The diagnosis and classification of asthma exacerbation severity in children are based on clinical assessment, including respiratory rate, level of consciousness, speech, use of accessory muscles, heart rate, oxygen saturation, and peak expiratory flow (PEF). These parameters help classify severity into mild/moderate, severe, and life-threatening categories. The criteria are presented in Tables 1 and 2 . 6

**Table 1:** Criteria for severity of asthma exacerbation in children (GINA, 2025)

Age range	Parameter	Mild/moderate	Severe/life-threatening
**6–11 years**	Body position	Sitting > lying	Sits hunched forwards
	Speech	Sentences	Words
	Level of consciousness	Not agitated	Agitated, drowsy, confused
	Respiratory rate	Increased	>30/min
	Accessory muscle use	No	Yes (paradoxical breathing)
	Pulse rate	100–120 bpm	>120 bpm or bradycardia
	SpO _2_ (on air)	90–95%	<90% (often markedly low)
	PEF (% predicted/best)	>50%	≤50% Not measurable/minimal
**Under 5 years**	Consciousness	Normal	Agitated, drowsy, or confused
	SaO _2_	>94%	<92%
	Speech/feeding	Sentences/normal feeding	Words/unable to speak or drink
	Respiratory rate	≤40/min	>40/min
	Accessory muscle use	No	Yes
	Pulse rate	<100/min	>180/min (0–3 years), >150/min (4–5 years)
	Central cyanosis	No	Present
	Wheeze intensity	Variable	Silent chest (no wheeze)

**Table 2:** Categorical risk factor descriptions for total sample and one-sample proportion test results

Variable	*n* (%) Category = “No”	*n* (%) Category = “Yes”	*n* (%) Missing	*χ*^2^ ( *p* )
Gender	Female = 77 (40.53)	Male = 113 (59.47)	–	6.62 (0.01)
Having fever	131 (68.95)	59 (31.05)	–	26.09 (<0.01)
History of hospital admission	78 (41.05)	112 (58.95)	–	5.89 (0.02)
History of PICU admission	155 (81.58)	35 (18.42)	–	73.71 (<0.01)
Lungs' crackles	151 (79.47)	39 (20.53)	–	64.10 (<0.01)
Lungs' reduced air entry	151 (79.47)	39 (20.53)	–	64.10 (<0.01)
Lungs' wheeze	19 (10)	171 (90)	–	120.48 (<0.01)
Magnesium sulfate used during hospitalization	157 (82.63)	33 (17.37)	–	78.77 (<0.01)
Obesity (BMI)	174 (91.58)	16 (8.42)	–	128.57 (<0.01)
On prophylactic medications	114 (60)	76 (40)	–	6.99 (0.01)
Passive smoking at home	171 (90)	19 (10)	–	118.90 (<0.01)
Past year asthma attacks	36 (18.95)	154 (81.05)	–	72.47 (<0.01)
Patient needed mechanical ventilation during hospitalization	179 (94.21)	11 (5.97)	–	145.52 (<0.01)
Severity assessment	56 (29.47)	134 (70.53)	–	31.53 (<0.01)
Systemic steroid started on admission	13 (6.84)	177 (93.16)	–	140.33 (<0.01)
Admitted to pediatric ward	18 (9.47)	172 (90.53)	–	123.69 (<0.01)
Admitted to PICU	159 (83.68)	31 (16.32)	–	84.00 (<0.01)
Allergic rhinoconjunctivitis	144 (75.79)	40 (21.05)	6 (3.16)	56.94 (<0.01)
Atopy	115 (60.53)	59 (31.05)	16 (8.42)	17.02 (<0.01)
Chest recession	59 (31.05)	131 (68.95)	–	26.84 (<0.01)
Consolidation on the chest X-ray study	156 (82.11)	29 (15.26)	5 (2.63)	84.91 (<0.01)
Cough	10 (5.26)	180 (94.74)	–	150.81 (<0.01)
Distress	34 (17.89)	156 (82.11)	–	77.49 (<0.01)
Family history of allergic disease	129 (67.89)	59 (31.05)	2 (1.05)	24.89 (<0.01)
Family history of asthma	90 (47.37)	100 (52.63)	–	0.47 (0.49)
Frequent need of systemic steroids	109 (57.37)	72 (37.89)	9 (4.47)	6.94 (0.01)

Abbreviation: PICU, pediatric intensive care unit.

### Statistical Methods

Cases of asthma and asthma exacerbations which presented at AQWCH between January 2018 and July 2023 were retrospectively identified using the Pediatric Department's electronic database. The study included 190 children aged under 13 years who were diagnosed with asthma exacerbation at admission, as defined by the GINA guidelines. 6 Obesity was defined as a body mass index (BMI) ≥ 30 kg/m ^2^ . 19 Patients were identified through a computerized search of hospital discharge diagnoses coded as “asthma,” “asthma exacerbation,” and “asthma attack.”

The primary aim was to develop a descriptive dataset of patients admitted with asthma during the study period. Secondary aims included examining patient characteristics and factors associated with asthma exacerbations, recognizing their clinical presentations, improving knowledge of their management, assessing adherence to prophylactic treatment, and evaluating patient outcomes.

Data abstractors were not involved in the study design or protocol development, which helped reduce observer bias.

### Statistical Analysis

#### Data Preprocessing

Data preprocessing included screening for missing values and assessing deviations from normality. Variables with a high proportion of missing data (e.g., total IgE and compliance with prophylactic medication, each with >70% missingness) were excluded from the final analysis.

For numerical variables with minimal missing data, mean imputation was applied. For categorical variables with minimal missingness (<5%), case-wise deletion was used. Categorical variables with very small sample sizes in one category (e.g., intravenous salbutamol use [ *n* = 4] and bronchiectasis [ *n* = 2]) were also excluded.

A full list of excluded variables is provided in the **Appendix** . The final dataset included 26 categorical and 10 numerical variables as potential risk factors.

Patients were classified into two groups—mild/moderate and severe/life-threatening—based on GINA (2025) criteria for children under 11 years. Asthma severity was used as the outcome variable.

#### Descriptive Analyses

Descriptive statistics included means, standard deviations, skewness, and kurtosis for numerical variables, and frequency distributions for categorical variables. Normality was assessed using |skewness| ≤ 3 and |kurtosis| ≤ 7, as recommended by Hair et al. (2010). 20 The number and proportion of cases in each severity group were also reported.

#### Preliminary Analyses

Preliminary analyses examined the distribution of categorical variables within the study population. One-sample proportion tests using Pearson's chi-square statistic were conducted to compare observed frequencies of each categorical variable against expected values and identify significantly more prevalent categories within the study population. All analyses were conducted with a significance threshold set at *p* < 0.05.

Associations between categorical variables and asthma severity were assessed using chi-square tests. For 2 × 2 tables with expected cell counts ≤ 5, Fisher's exact test was used.

For continuous variables, independent samples *t* -tests were applied when normality assumptions were met. Homogeneity of variances was assessed using the F-test, and the Welch correction was applied where variances were unequal. For non-normally distributed variables, the Mann-Whitney U test was used. Details of normality and homogeneity of variance checks are provided in the **Appendix** .

#### Logistic Regression Analyses

Logistic regression analyses were conducted to evaluate the association between risk factors and asthma severity. Given the binary outcome, models with a binomial distribution were fitted, with asthma severity as the dependent variable and individual risk factors as independent variables. Odds ratios (ORs) with 95% confidence intervals (CIs) were reported.

A backward stepwise selection approach was used in multiple logistic regression to identify significant predictors for the final model. The method begins with a full model including all potential predictors and iteratively removes non-significant predictors based on *p* -values or criteria such as Akaike information criterion (AIC) and Bayesian information criterion (BIC), until only those predictors that contribute meaningfully to the model remain.

For all analyses, “mild/moderate” severity and “No” for categorical predictors were used as reference categories. Statistical significance was set at *p* < 0.05. All analyses were conducted using R software (version 4.4.2).

## Results

### Demographic and Clinical Characteristics

A summary of the demographic and clinical characteristics for the overall sample, along with the results of one-sample proportion tests, is presented in Table 2 . The mean age of the patients was 5.34 years (SD = 2.65), and the mean length of stay was 2.54 days (SD = 2.08).

Of the total sample, 113 patients were male and 77 were female. A significantly higher proportion of males was observed (χ ^2^ (1) = 6.62, *p* = 0.01).

A total of 31 patients (16.32%) were admitted to the PICU (χ ^2^ (1) = 84.00, *p* < 0.01), while 172 patients (90.53%) were admitted to the pediatric ward (χ ^2^ (1) = 123.69, *p* < 0.01).

Regarding long-term management, 40% of patients were receiving prophylactic medications (χ ^2^ (1) = 6.99, *p* = 0.01). In the past year, 81.05% had experienced at least one asthma attack exacerbation (χ ^2^ (1) = 72.47, *p* < 0.01). A history of hospital admission was reported in 58.95% of patients (χ ^2^ (1) = 5.89, *p* = 0.02), whereas 18.42% ( *n* = 35) had a history of PICU admission (χ ^2^ (1) = 73.71, *p* < 0.01).

Most patients were assessed as having severe asthma exacerbation (70.53%, χ ^2^ (1) = 31.53, *p* < 0.01). A very high proportion (93.16%) received systemic steroids upon admission (χ ^2^ (1) = 140.33, *p* < 0.01), and 37.89% reported a frequent need for systemic steroids (χ ^2^ (1) = 6.94, *p* = 0.01). Additionally, 31.05% of patients reported a family history of allergic disease (χ ^2^ (1) = 24.89, *p* < 0.01). In contrast, family history of asthma was nearly equally distributed between “yes” and “no” (χ ^2^ (1) = 0.47, *p* = 0.49), suggesting no clear predominance. Exposure to passive smoking at home was reported in 10% of patients (χ ^2^ (1) = 118.90, *p* < 0.01).

Most patients did not present with fever (68.95%) (χ ^2^ (1) = 26.09, *p* < 0.01). Chest recession was reported in 68.95% of cases (χ ^2^ (1) = 26.84, *p* < 0.01), while cough was present in 94.74% of patients (χ ^2^ (1) = 150.81, *p* < 0.01). Distress was also highly prevalent, as it was observed in 82.11% of patients (χ ^2^ (1) = 77.49, *p* < 0.01).

Most patients did not exhibit lung crackles or reduced air entry (χ ^2^ (1) = 64.10, *p* < 0.01), whereas lung wheeze was present in 90% of patients (χ ^2^ (1) = 120.48, *p* < 0.01).

Clinical examination findings included a mean heart rate of 141.32 beats per minute (SD = 21.31) and a mean respiratory rate of 42.79 breaths per minute (SD = 11.09). No patients had a personal or family history of immunodeficiency, cystic fibrosis, failure to thrive, or dullness on clinical examination.

### Radiologic, Hematologic, and Microbiologic Findings Upon Admission

Radiological findings at the time of admission were recorded. Chest X-rays were obtained for 74 patients (39%), revealing consolidation in 15.26% of patients (χ ^2^ (1) = 84.91, *p* < 0.01) and pleural effusion in 5 patients (2.63%).

Blood tests conducted upon admission revealed a mean WBC count of 13.34 (SD = 5.03), a mean hemoglobin level of 12.19 g/dL (SD = 1.21), and a mean MCV of 75.8 fL (SD = 6.56). The mean CRP level was 17.43 mg/L (SD = 23.69).

### Management, Associated Comorbidities, and Follow-up

Of the total patients, 177 (93.16%) received systemic steroids upon admission (χ ^2^ (1) = 140.33, *p* < 0.01), and 37.89% reported a frequent need for systemic steroids in the past year (χ ^2^ (1) = 6.94, *p* = 0.01).

During hospitalization, 33 patients (17.37%) were treated with magnesium sulfate (χ ^2^ (1) = 78.77, *p* < 0.01), 4 patients (2.10%) received intravenous salbutamol, and mechanical ventilation was rarely required (χ ^2^ (1) = 145.52, *p* < 0.01).

The most common associated comorbidities included atopy in 31.05% of patients (χ ^2^ (1) = 17.02, *p* < 0.01). Allergic rhinoconjunctivitis was present in 21.05% of patients (χ ^2^ (1) = 56.94, *p* < 0.01). Most children were not obese (91.58%, χ ^2^ (1) = 128.57, *p* < 0.01). Other conditions included gastroesophageal reflux (GER) in 10 cases (5.26%), obstructive sleep apnea (OSA) in 6 (3.16%), rhinosinusitis in 6 (2.63%), and bronchiectasis in 2 (1.05%).

A total of 78 patients (41%) attended follow-up appointments, and spirometry was performed for 40 patients (21.05%).

### Association of Demographic and Clinical Characteristics with Asthma Exacerbation

Table 3 presents the chi-square and Fisher's exact tests conducted on the categorical risk factors. Among these factors, obesity was significantly associated with severe asthma (OR = 0.19, *p* = 0.02), indicating that obese patients are less common in the severe/life-threatening group than in the mild/moderate group.

**Table 3:** Categorical risk factor descriptions by groups and chi-square and Fisher's exact tests of association

		Mild/moderate ( *N* = 113)	Severe/life-threatening ( *N* = 77)	
Variable	Category	*n* (%)	*n* (%)	*χ*^2^ ( *p* )/OR ( *p* )
Gender	Female	45 (23.68)	32 (16.84)	*χ*^2^ = 0.01 (0.93)
	Male	68 (35.79)	45 (23.68)	
Having fever	No	75 (39.47)	56 (29.47)	*χ*^2^ = 0.59 (0.44)
	Yes	38 (20)	21 (11.05)	
History of hospital admission	No	49 (25.79)	29 (15.26)	*χ*^2^ = 0.40 (0.53)
	Yes	64 (33.68)	48 (25.26)	
History of PICU admission	No	94 (49.47)	61 (32.11)	*χ*^2^ = 0.25 (0.62)
	Yes	19 (10)	16 (8.42)	
Lungs' crackles	No	92 (48.42)	59 (31.05)	*χ*^2^ = 0.38 (0.54)
	Yes	21 (11.05)	18 (9.47)	
Lungs' reduced air entry	No	87 (45.79)	64 (33.68)	*χ*^2^ = 0.71 (0.40)
	Yes	26 (13.68)	13 (6.84)	
Lungs' wheeze	No	15 (7.89)	4 (2.11)	OR = 2.78 (0.09)
	Yes	98 (51.58)	73 (38.42)	
Magnesium sulfate used during hospitalization	No	96 (50.53)	61 (32.11)	*χ*^2^ = 0.69 (0.41)
	Yes	17 (8.95)	16 (8.42)	
Obesity (BMI)	No	99 (52.11)	75 (39.47)	OR = 0.19 (0.02)
	Yes	14 (7.37)	2 (1.05)	
On prophylactic medications	No	76 (40)	38 (20)	*χ*^2^ = 5.39 (0.02)
	Yes	37 (19.47)	39 (20.53)	
Passive smoking at home	No	102 (53.68)	69 (36.32)	*χ*^2^ = 0.00 (0.99)
	Yes	11 (5.79)	8 (4.21)	
Past year asthma attacks	No	25 (13.16)	11 (5.79)	*χ*^2^ = 1.36 (0.24)
	Yes	88 (46.32)	66 (34.74)	
Patient needed mechanical ventilation during hospitalization	No	107 (56.32)	72 (37.89)	OR = 1.24 (0.76)
	Yes	6 (3.16)	5 (2.63)	
Severity assessment	No	29 (15.26)	27 (14.21)	*χ*^2^ = 1.52 (0.22)
	Yes	84 (44.21)	50 (26.32)	
Systemic steroid started on the admission	No	11 (5.79)	2 (1.05)	OR = 4.02 (0.08)
	Yes	102 (53.68)	75 (39.47)	
Admitted to pediatric ward	No	10 (5.26)	8 (4.21)	*χ*^2^ = 0.01 (0.92)
	Yes	103 (54.21)	69 (36.32)	
Admitted to PICU	No	95 (50)	64 (33.68)	*χ*^2^ = 0.00 (0.99)
	Yes	18 (9.47)	13 (6.84)	
Allergic rhinoconjunctivitis	No	87 (45.79)	57 (30)	*χ*^2^ = 0.00 (0.99)
	Yes	24 (12.63)	16 (8.42)	
	Missing	2 (1.05)	4 (2.11)	
Atopy	No	76 (40)	39 (20.53)	*χ*^2^ = 3.19 (0.07)
	Yes	30 (15.79)	29 (15.26)	
	Missing	7 (3.68)	9 (4.74)	
Chest recession	No	42 (22.11)	17 (8.95)	*χ*^2^ = 4.19 (0.04)
	Yes	71 (37.37)	60 (31.58)	
Consolidation on the chest X-ray study	No	94 (49.47)	62 (32.63)	*χ*^2^ = 0.43 (0.51)
	Yes	15 (7.89)	14 (7.37)	
	Missing	4 (2.11)	1 (0.53)	
Cough	No	7 (3.68)	3 (1.58)	OR = 1.62 (0.74)
	Yes	106 (55.79)	74 (38.95)	
Distress	No	27 (14.21)	7 (3.68)	*χ*^2^ = 5.86 (0.02)
	Yes	86 (45.26)	70 (36.84)	
Family history of allergic disease	No	85 (44.74)	44 (23.16)	*χ*^2^ = 6.00 (0.01)
	Yes	27 (14.21)	32 (16.84)	
	Missing	1 (0.53)	1 (0.53)	
Family history of asthma	No	59 (31.05)	31 (16.32)	*χ*^2^ = 2.17 (0.14)
	Yes	54 (28.42)	46 (24.21)	
Frequent need of systemic steroids	No	66 (34.74)	43 (22.63)	*χ*^2^ = 0.01 (0.91)
	Yes	45 (23.68)	27 (14.21)	
	Missing	2 (1.05)	7 (3.68)	

Abbreviation: PICU, pediatric intensive care unit.

Use of prophylactic medications was significantly associated with asthma severity (χ ^2^ = 5.39, *p* = 0.02), with a higher proportion of patients on prophylactic medications in the severe/life-threatening group. Chest recession was also significantly associated with asthma severity (χ ^2^ = 4.19, *p* = 0.04), being more frequently observed in the severe/life-threatening group.

A significant association was observed between distress and asthma severity (χ ^2^ = 5.86, *p* = 0.02), with distress being more common among patients with severe/life-threatening asthma. Family history of allergic disease was also significantly associated with asthma severity (χ ^2^ = 6.00, *p* = 0.01), indicating that patients with such a history were more likely to experience severe/life-threatening asthma.

### Differences in Numerical Risk Factors Between the Two Patient Groups

Comparisons between mild/moderate and severe/life-threatening asthma groups using two-sample *t* -tests and Mann-Whitney U tests are presented in Table 4 .

**Table 4:** Comparisons of numerical risk factors between two groups of patients

	Total ( *N* = 190)	Mild/moderate ( *N* = 113)	Severe/life-threatening ( *N* = 77)		
Variable	Mean (SD)	Mean (SD)	Mean (SD)	Test statistic ( *p* )	Effect size
Length of stay	2.54 (2.08)	2.77 (2.41)	2.21 (1.44)	*U* = 4713 (0.31)	0.08
Age	5.34 (2.65)	6.04 (2.83)	4.32 (1.99)	*t* = 4.90 (<0.01)	0.70
Respiratory rate	42.79 (11.09)	39.01 (10.72)	48.35 (9.16)	*t* = −6.25 (<0.01)	0.92
Heart rate	141.32 (21.31)	137.75 (22.45)	146.56 (18.42)	*t* = −2.85 (<0.01)	0.42
Oxygen saturation	94.03 (4.44)	95.65 (3.06)	91.65 (5.06)	*t* = 6.20 (<0.01)	0.95
WBCA	13.34 (5.03)	12.97 (5.08)	13.88 (4.93)	*t* = −1.23 (0.22)	0.18
Hemoglobin	12.19 (1.21)	12.26 (1.26)	12.08 (1.13)	*t* = 1.02 (0.31)	0.15
Mean corpuscular volume	75.8 (6.56)	75.37 (6.74)	76.42 (6.27)	*t* = −1.07 (0.28)	0.16
Absolute eosinophils	357.5 (377.98)	361.06 (376.58)	352.27 (382.43)	*U* = 4457.5 (0.77)	0.02
CRPb	17.43 (23.69)	17.09 (22.56)	17.93 (25.4)	*U* = 4168.5 (0.63)	0.04

Abbreviations: t, t-test statistic; U, Mann-Whitney statistic; WBCA, white blood cell count; CRPb, C-reactive protein binding.

Among the numerical risk factors, age differed significantly between the two groups ( *t* = 4.90, *p* < 0.01), with a medium effect size of *d* = 0.70. Children in the severe/life-threatening group were younger than those in the mild/moderate group.

A significant difference was also observed in respiratory rate ( *t* = −6.25, *p* < 0.01), with a large effect size of *d* = 0.92 with higher values in the severe/life-threatening group. The comparison between the two groups in terms of heart rate revealed a significant difference ( *t* = −2.85, *p* < 0.01), with a small effect size of *d* = 0.42. The severe/life-threatening group had a higher mean heart rate than the mild/moderate group. Furthermore, there was a significant difference between the two groups in terms of oxygen saturation ( *t* = 6.20, *p* < 0.01), with a large effect size of *d* = 0.95. Oxygen saturation was significantly lower in the severe/life-threatening group compared with the mild/moderate group.

None of the remaining numerical factors showed significant differences between the two groups.

### Association of Risk Factors with the Odds of Severe/Life-threatening Asthma

The association of each risk factor with the odds of severe asthma was assessed using simple logistic regression models. The results are reported in Table 5 .

**Table 5:** Simple logistic results of patients with mild/moderate asthma as the reference group

Variable	Estimate	Std. error	*p* -value	OR	95% CI
Gender (male)	−0.07	0.30	0.81	0.93	(0.52, 1.68)
Having fever	−0.30	0.32	0.35	0.74	(0.39, 1.39)
Cough	0.49	0.71	0.49	1.63	(0.44, 7.75)
Distress	1.14	0.45	0.01	3.14	(1.35, 8.21)
Chest recession	0.74	0.34	0.03	2.09	(1.09, 4.12)
Lungs' wheeze	1.03	0.58	0.08	2.79	(0.97, 10.11)
Lungs' reduced air entry	−0.39	0.38	0.31	0.68	(0.32, 1.4)
Lungs' crackles	0.29	0.36	0.42	1.34	(0.65, 2.72)
Severity assessment	−0.45	0.32	0.16	0.64	(0.34, 1.2)
Admitted to PICU	0.07	0.40	0.86	1.07	(0.48, 2.33)
Admitted to pediatric ward	−0.18	0.50	0.72	0.84	(0.31, 2.29)
On prophylactic medications	0.75	0.30	0.01	2.11	(1.17, 3.84)
Past year asthma attacks	0.53	0.40	0.18	1.70	(0.8, 3.84)
Frequent need of systemic steroids	−0.08	0.31	0.79	0.92	(0.5, 1.7)
History of hospital admission	0.24	0.30	0.43	1.27	(0.7, 2.3)
Family history of asthma	0.48	0.30	0.11	1.62	(0.91, 2.93)
Family history of allergic disease	0.83	0.32	0.01	2.29	(1.23, 4.32)
Passive smoking at home	0.07	0.49	0.88	1.08	(0.4, 2.79)
History of PICU admission	0.26	0.38	0.49	1.30	(0.61, 2.72)
Consolidation on the chest X-ray study	0.35	0.41	0.39	1.42	(0.63, 3.15)
Systemic steroid started on the admission	1.40	0.78	0.08	4.04	(1.05, 26.63)
Magnesium sulfate used during hospitalization	0.39	0.39	0.31	1.48	(0.69, 3.16)
Patient needed mechanical ventilation during hospitalization	0.21	0.62	0.73	1.24	(0.35, 4.26)
Allergic rhinoconjunctivitis	0.02	0.37	0.96	1.02	(0.49, 2.07)
Obesity (BMI)	−1.67	0.77	0.03	0.19	(0.03, 0.7)
Atopy	0.63	0.33	0.05	1.88	(0.99, 3.59)
Length of stay	−0.15	0.08	0.07	0.86	(0.72, 1)
Age	−0.28	0.07	<0.01	0.75	(0.66, 0.86)
Respiratory rate	0.09	0.02	<0.01	1.09	(1.06, 1.14)
Heart rate	0.02	0.01	0.01	1.02	(1.01, 1.04)
Oxygen saturation	−0.24	0.04	<0.01	0.79	(0.72, 0.85)
WBCA	0.04	0.03	0.22	1.04	(0.98, 1.1)
Hemoglobin	−0.13	0.12	0.31	0.88	(0.69, 1.12)
Mean corpuscular volume	0.03	0.02	0.28	1.03	(0.98, 1.07)
Ln (absolute eosinophils)	−0.06	0.14	0.65	0.94	(0.72, 1.23)
Ln (CRPb)	0.05	0.10	0.59	1.06	(0.87, 1.29)

Abbreviation: PICU, pediatric intensive care unit; WBCA, white blood cell count; CRPb, C-reactive protein binding.

Note: *Ln* represents natural logarithmic transformation. For categorical variables, the reference category is “No,” and for gender, it is “Female.”

The simple logistic regression analyses showed that children experiencing distress had significantly higher odds of severe/life-threatening asthma (OR = 3.14, 95% CI = [1.35, 8.21]). Children who presented with distress were over three times more likely to have a severe/life-threatening asthma exacerbation compared with those without distress. Chest recession was also associated with increased odds of severe/life-threatening asthma (OR = 2.09, 95% CI = [1.09, 4.12]). The use of prophylactic medications was linked to greater odds of severe/life-threatening asthma (OR = 2.11, 95% CI = [1.17, 3.84]), indicating that children who require prophylactic medications may be at greater risk for severe attacks. Having a family history of allergic disease significantly increased the odds of severe asthma among the children (OR = 2.29, 95% CI = [1.23, 4.32]).

Obesity was associated with lower odds of severe/life-threatening asthma (OR = 0.19, 95% CI = [0.03, 0.70]), suggesting that obese children in this sample were less likely to experience the most severe exacerbations.

Higher age was associated with lower odds (OR = 0.75, 95% CI = [0.66, 0.86]), indicating that younger children were at higher risk. Increased respiratory rate was associated with higher odds (OR = 1.09, 95% CI = [1.06, 1.14]). For each unit increase in respiratory rate, the odds of severe/life-threatening asthma increased by 9%. Higher heart rate was also associated with greater odds of severe/life-threatening asthma (OR = 1.02, 95% CI = [1.01, 1.04]).

Higher oxygen saturation was protective, with each percentage point increase associated with a 21% reduction in the odds of severe/life-threatening asthma (OR = 0.79, 95% CI = [0.72, 0.85]).

### Multiple Logistic Regression Results

In the final multiple logistic regression model determined by backward selection ( Table 6 ), several risk factors remained significantly associated with the odds of severe/life-threatening asthma.

**Table 6:** Multiple logistic regression results predicting odds of severe asthma

Variable	Estimate	Std. error	*p* -value	OR	95% CI
Having fever	−0.93	0.58	0.11	0.39	(0.12, 1.2)
Distress	0.82	0.75	0.28	2.26	(0.54, 10.57)
Severity assessment	−0.82	0.55	0.13	0.44	(0.15, 1.27)
Admitted to pediatric ward	0.72	1.01	0.48	2.04	(0.3, 16.01)
On prophylactic medications	−0.08	0.58	0.90	0.93	(0.29, 2.88)
Past year asthma attacks	1.29	0.69	0.06	3.62	(0.98, 15.31)
History of hospital admission	−0.95	0.61	0.12	0.39	(0.11, 1.25)
Magnesium sulfate used during hospitalization	1.39	0.77	0.07	4.00	(0.93, 19.08)
Obesity (BMI)	−1.93	1.29	0.13	0.15	(0.01, 1.5)
Atopy	−0.65	0.59	0.27	0.52	(0.16, 1.63)
Length of stay	−0.25	0.13	0.06	0.78	(0.59, 0.99)
Age	−0.58	0.16	<0.01	0.56	(0.4, 0.75)
Respiratory rate	0.06	0.03	0.04	1.06	(1, 1.13)
Heart rate	−0.01	0.02	0.44	0.99	(0.96, 1.02)
Oxygen saturation	−0.50	0.10	<0.01	0.60	(0.49, 0.72)
Hemoglobin	−0.29	0.26	0.26	0.75	(0.45, 1.23)
Mean corpuscular volume	0.07	0.04	0.11	1.07	(0.99, 1.17)

Note: * *p* < 0.05; ** *p* < 0.01. *Ln* represents natural logarithmic transformation.

Older age was associated with lower odds (OR = 0.56, 95% CI = [0.40, 0.75]), indicating that younger children were more likely to experience severe exacerbations. Higher respiratory rate was associated with increased odds (OR = 1.06, 95% CI = [1.00, 1.13]), with each unit increase raising the odds by 6%.

Higher oxygen saturation was associated with reduced odds (OR = 0.60, 95% CI = [0.49, 0.72]), with each percentage point increase corresponding to a 40% reduction in odds.

## Discussion

Asthma exacerbation is one of the most common causes of emergency visits and hospital admissions among children with asthma. This research aimed to explore potential causes and identifiable risk factors associated with worsening asthma symptoms that may lead to severe exacerbations. This understanding can help parents seek timely medical care and initiate early, appropriate management.

Among the identified risk factors were exposure to allergens, environmental smoke, dietary influences, cold weather, physical activity, recent hospital or emergency department visits for asthma, excessive use of inhaled bronchodilators, incorrect inhaler usage, obesity, the presence of eosinophils in sputum or blood, additional health conditions, the use of aspirin and other NSAIDs, ineffective asthma management, poor treatment adherence, and low socioeconomic status. Additionally, admission to intensive care, requiring intubation, and discontinued medical care were categorized as risk factors. 19

In our study, we analyzed risk factors for asthma exacerbations. We found that they were more common in males and younger age groups. Research did not definitively establish whether exacerbation rates are higher in males or females; some studies found a higher percentage among females, while others reported more males. 21 22 23 In line with other studies, having an asthma attack in the past year is a significant risk factor for future exacerbations. 6 24 Additionally, frequent use of steroids has been identified as a risk factor for exacerbations, consistent with prior research. 25 26 27 A history of hospital admissions can also indicate a potential for future attacks if a child's asthma is not properly managed. Our study revealed a higher risk of asthma attacks in those with previous hospital admissions, which aligns with international findings. 6 24 Prophylactic medication is important to avoid exacerbation of asthma and improve children's quality of life. Further, our study showed that children not on prophylactic medication are at greater risk of developing asthma exacerbation, similar to existing literature. 6 28

Smoking exposure is widely recognized as a major risk factor affecting respiratory health, with particular effect on the lungs, as numerous studies have demonstrated. 29 Our research found a lower incidence compared with these studies, which may be due to parents not responding accurately or the presence of designated smoking areas at homes. In the present study, most patients did not have fever, which explains the fact that most asthma exacerbations are triggered by other factors.

Allergic conditions, including atopy and allergic rhinitis, have been documented in various journals to increase the likelihood of asthma attacks. 30 In our study, allergies and atopic conditions were shown to be significant risk factors for worsening asthma. Obesity is another factor known to complicate asthma management and increase severity. Obese patients often exhibit reduced responsiveness to ICS, altered lung function, and more frequent emergency department visits. 31 Similarly in the present study, obesity was identified as a risk factor. This study highlights a particularly significant distinction—unlike other research, a notable percentage of patients had previously been admitted to the PICU. 32

We then proceeded with an analysis of logistic regression to identify variables associated with mild/moderate and severe/life-threatening exacerbations. We found that younger age, obesity, chest recession, higher heart and respiratory rates, patient distress, family history of atopy, being on prophylactic medication, and lower oxygen saturation were significant risk factors for developing severe asthma exacerbation.

Approximately 70% of children in our study experienced severe asthma exacerbations, characterized by respiratory distress, chest retraction, tachypnea, and tachycardia. These findings are well-recognized clinical indicators of severe asthma exacerbation and can be explained by airway obstruction and increased work of breathing.

Upon admission, the use of administration of systemic steroids and magnesium sulfate serves as indirect signs of management for moderate to severe asthma exacerbations, as these treatments are reserved for escalation therapy. Acute exacerbations were identified by the administration of systemic steroids and magnesium sulfate upon admission. While wheezing and cough were present in all patients, these clinical findings in our study were consistent with those published in the literature. 6 33

Normal oxygen saturation in children is ≥94%. If saturation falls below this threshold (hypoxemia), immediate management should begin with the administration of oxygen via a cannula or face mask. To prevent the development of respiratory failure, titrated oxygen should be provided. Oxygen saturation is a crucial indicator of severity, reflecting airway responsiveness (with obstruction indicated by levels below 94%) and the adequacy of gas exchange. Oxygen saturation below 92% is associated with severe asthma exacerbation and the necessity for hospitalization. 6 34 35 These variables are reliable predictors of severity, as demonstrated in our study.

Age-related risk factors are more prevalent in younger patients, which can be attributed to smaller airway diameters, increased susceptibility to viral triggers, and reduced anatomical reserve. Similar to our study, Engelsvold and Oymar reported a strong correlation between severe attacks and younger age. 26

Adherence to ICS is essential for effective asthma control. Compliance with prophylactic medications is essential, as it can reduce future exacerbations and minimize direct and indirect costs, such as hospital stays and emergency visits, while improving the patient's quality of life through enhanced daily activities and sports participation. 8 27 A high medication load in general is a sign of poor asthma control, particularly poor medication compliance and increased disease severity. Interestingly, our study revealed an increase in the odds of severe exacerbation in patients on prophylactic medications. This incidence is consistent with confounding by indication, as step-up therapy itself serves as a marker of risk for severe exacerbations. 26

The mechanisms by which allergens trigger asthma exacerbations are well-studied, involving IgE-mediated inflammation, eosinophilia, and the release of inflammatory cytokines that promote airway inflammation and bronchial mucus hypersecretion in asthma. Allergies, including a family history of allergy, atopy, and allergic rhinitis, have been documented in various journals to increase the likelihood of asthma attacks. 30 In our study, we also found that a family history of allergy and atopy are significant risk factors for severe exacerbations, suggesting that children with an allergic background have a higher incidence of developing severe exacerbations.

Obesity is another contributor that complicates asthma management and exacerbates the severity of attacks. Obese patients often show reduced responsiveness to ICS, altered lung function, and more frequent emergency department visits. An international study indicated a slightly increased association between asthma attacks and obesity. 23 36 Interestingly, in our study, obese patients were less prone to developing severe asthma exacerbations. We hypothesize that obese children may exert little to no effort, and their symptoms may manifest more quickly than those of other patients, leading to more frequent visits to healthcare facilities and earlier treatment. 37

Fewer than half of the patients attended follow-up appointments. We theorize that this low attendance rate may be attributed to either a lack of knowledge about asthma or financial constraints. Therefore, we emphasize the importance of raising awareness about asthma, not only among patients and their families but also among healthcare professionals and general practitioners.

The strength of this study lies in its status as the first investigation to evaluate these causes within the pediatric populations in the country, providing crucial data for future research. The results of our study have significant clinical and practical implications. Early detection of high-risk patients based on quick and simple clinical signs (such as distress and chest retractions) and vital signs (including respiratory rate and oxygen saturation) can facilitate rapid triage. Preventative and prophylactic management can be tailored for children with allergic backgrounds and younger ages, helping to avert severe or life-threatening asthma exacerbations.

However, this study has several limitations as well. We were unable to locate or may have overlooked documentation for certain variables (e.g., total IgE and compliance with prophylactic medication) in the patients' medical records. In this study, several risk factors were not significantly associated with asthma exacerbation severity, including gender, fever, prior hospital or PICU admissions, passive smoking, and laboratory parameters. This may be explained by the acute clinical presentation, which may be more predictive of severity than historical or laboratory findings. Additionally, because the research was conducted at a single center, the findings may not be representative of the entire country. Therefore, a prospective study in multicenter setting is needed to provide more comprehensive data on asthma in the country.

## Conclusion

Numerous studies have been conducted to identify the risk factors contributing to asthma exacerbations. In this study, we performed multivariate logistic regression analysis to identify variables associated with mild/moderate and severe/life-threatening exacerbations. We found that patients exhibiting distress, increased respiratory and heart rates, a family history of allergy, lower oxygen saturation, and use of prophylactic medications had a higher risk of developing severe asthma exacerbations. Conversely, obese children had a lower probability of experiencing severe exacerbations.

Evaluating these factors can aid clinicians in managing asthma more effectively. However, there is no definitive guidance on which specific risk factors, or under what circumstances, may lead to exacerbations. Identifying potential triggers can assist patients if they are advised on how to avoid them.

Despite these efforts, significant work remains to be done both clinically and in public health. Educating the public about asthma is essential. Further multicenter research may uncover additional risks not addressed in this study.

## Figures and Tables

**Table A1:** Variables excluded from the study due to a high proportion of missingness

	Count	Percentage
Complaint to prophylactic medications	112	58.95
Family history of cystic fibrosis	84	44.21
Family history of failure to thrive	85	44.74
Total IgE	147	77.37
Vitamin D level	158	83.16
Iron level	157	82.63
Any detected pathogen in viral studies	170	89.47
Ferritin level	160	84.21
Transferrin level	175	92.11
Total iron binding capacity	184	96.84
Gastroesophageal reflux	68	35.79

**Table A2:** Variables excluded from the study due to a small number in one category

Variable	Category	Count	Percentage
Bronchiectasis	Yes	2	1.05
	No	188	98.95
IV salbutamol used during hospitalization	Yes	4	2.11
	No	186	97.89
Obstructive sleep apnea	Yes	6	3.16
	No	184	96.84
Pleural effusion on the chest X-ray study	Yes	5	2.63
	No	180	94.74
	Missing	5	2.63
Rhinosinusitis nasal polyposis	Yes	5	2.63
	No	180	94.74
	Missing	5	2.63

**Table A3:** Distributional measures of numerical risk factors

	Total ( *N* = 190)		Mild/moderate ( *N* = 113)		Severe/life-threatening ( *N* = 77)		
Variable	Skewness	Kurtosis	Skewness	Kurtosis	Skewness	Kurtosis	Result
Length of stay	2.39	9.36	2.26	7.46	1.11	1.05	Non-normal
Age	0.64	−0.43	0.15	−0.77	1.64	2.36	Normal
Respiratory rate	0.35	0.06	0.73	0.85	0.37	0.16	Normal
Heart rate	−0.34	1.08	−0.37	1.21	0.09	−0.48	Non-normal
Oxygen saturation	−0.8	0.17	−0.34	−1.03	−0.28	−0.83	Non-normal
WBCA	0.72	0.73	0.59	0.57	0.96	0.79	Normal
Hemoglobin	0.12	1.12	−0.2	1.22	0.71	1.06	Normal
Mean corpuscular volume	−0.7	0.39	−0.68	−0.04	−0.69	1.05	Non-normal
Absolute eosinophils	2.09	5.74	2.16	6.9	1.96	3.93	Non-normal
CRPb	2.74	8.94	2.29	5.55	3.13	11.37	Non-normal

**Table A4:** Tests of homogeneity of variances between two groups of patients using Fisher's test

Variable	*F* ( *df*_1_ , *df*_2_ )	*p* -value		Result
Age	2.02 (112, 76)	<0.01		Unequal variances
Heart rate	1.49 (112, 76)	0.07		Equal variances
Hemoglobin	1.24 (112, 76)	0.31		Equal variances
Mean corpuscular volume	1.16 (112, 76)	0.50		Equal variances
Oxygen saturation	0.36 (112, 76)	<0.01		Unequal variances
Respiratory rate	1.37 (112, 76)	0.15		Equal variances
White blood cell count (WBCA)	1.06 (112, 76)	0.78		Equal variances

## Notes

**What Is Known About This Topic?**

- The burden of asthma exacerbations on healthcare services is increasing.
- Mortality and morbidity associated with asthma exacerbations are relatively common.
- Asthma medications are essential for effective management.

**What This Study Adds?**

- This study identified various risk factors for asthma exacerbations in children, such as younger age, male gender, previous asthma exacerbation, and past-year hospital admission.
- It is also demonstrated that risk factors for severe asthma exacerbations, according to asthma severity, include respiratory distress, chest recession, tachycardia, tachypnea, hypoxemia, previous hospital admissions, and patients who are on prophylactic medication.
- Asthma research to predict risk factors for severity is limited in the United Arab Emirates. Our study provides local data from the United Arab Emirates on risk factors for asthma exacerbations, adding to the local evidence base.

**How This Study Impacts Current Management Guidelines?**

- This study highlights the importance of identifying high-risk patients and adapting treatment accordingly. Such patients should be flagged for high alert when visiting healthcare facilities and require close follow-up and individualized management plans.
